# Efficacy and Safety of the Smallpox Vaccine for Postexposure Prophylaxis in Monkeypox: Protocol for an Open-Labeled, Single-Armed Study

**DOI:** 10.2196/46955

**Published:** 2023-08-25

**Authors:** Rina Yano, Junko Terada-Hirashima, Yukari Uemura, Noriko Tomita, Yosuke Shimizu, Haruka Iwasaki, Nobumasa Okumura, Tetsuya Suzuki, Sho Saito, Mugen Ujiie, Wataru Sugiura, Norio Ohmagari

**Affiliations:** 1 Center for Clinical Sciences National Center for Global Health and Medicine Tokyo Japan; 2 Disease Control and Prevention Center Center Hospital of the National Center for Global Health and Medicine Tokyo Japan

**Keywords:** monkeypox, post-exposure prophylaxis, smallpox vaccine, vaccinia virus, LC16, poxvirus, smallpox, vaccination, smallpox virus, orthopoxvirus, immunity, epidemiology, zoonosis, virus, vaccine, Disease Control and Prevention, inoculation, injection site, body temperature, headache, rash, lymphadenopathy, infectious disease, endpoint analysis

## Abstract

**Background:**

In May 2022, a case of monkeypox (currently known as “mpox”) with no history of overseas travel was reported in the United Kingdom, followed by reports of infections reported in Europe, the United States, and other countries worldwide. Due to the significant overlap in immune responses among viruses of the genus *Orthopoxvirus* (including smallpox virus, mpox virus, and vaccinia virus), it is believed that cross-immunity can be achieved by administering the smallpox virus vaccine. In Japan, a smallpox vaccine (LC16m8 strain vaccine) has been approved; however, there was no regulatory approval for the mpox vaccine during the design of this study. Although it is believed that individuals exposed to the mpox virus may receive smallpox vaccination as mpox prophylaxis, the existing evidence is not clear.

**Objective:**

The primary objective was to evaluate the efficacy of the LC16m8 strain vaccine, approved for smallpox in Japan, for postexposure prophylaxis against mpox when administered to close contacts of individuals with mpox. The secondary objective was to investigate the safety of the vaccine for postexposure prophylaxis against mpox.

**Methods:**

The study aimed to enroll 100 vaccinated participants who had been identified as close contacts of individuals with mpox. Consent was obtained, and the participants are inoculated with the vaccine. Daily recordings of symptoms (body temperature, headache, rash, and side effects) were made until day 21 and then again on day 28. Furthermore, additional evaluations of adverse events were performed by the investigators on days 7, 14, 21, and 28. Considering that the maximum incubation period for mpox is 21 days, the primary end point is the presence or absence of the disease 21 days after close contact. The primary analysis focused on cases within 4 days of intense contact as it has been reported that vaccination within this timeframe can reduce the incidence of the disease.

**Results:**

The first trial participant was enrolled on July 28, 2022, and the research period concluded in March 2023. The study results will be published in a peer-reviewed scientific journal.

**Conclusions:**

This study allowed us to investigate the efficacy and safety of the LC16m8 strain vaccine in postexposure prophylaxis against mpox.

**Trial Registration:**

Japan Registry of Clinical Trials jRCTs031220137; https://jrct.niph.go.jp/en-latest-detail/jRCTs031220137

**International Registered Report Identifier (IRRID):**

DERR1-10.2196/46955

## Introduction

Monkeypox is a zoonotic pathogenic virus belonging to the genus *Orthopoxvirus* of the family Poxviridae. It was initially isolated from a cynomolgus monkey in 1958 and was named monkeypox [1]. However, in November 2022, the World Health Organization (WHO) changed the name to “mpox” as its natural host is considered to be a rodent [2]. Mpox circulates within a transmission cycle involving rodents and monkeys in tropical rainforests spanning from Central Africa to West Africa [3]. Since the first confirmed human infection in the Democratic Republic of the Congo in 1970 [4-6], human infections have been reported following bites from virus-infected monkeys and rodents, as well as, through the ingestion of undercooked meat. Human-to-human transmission via droplets and direct contact has also been documented [3,7,8]. While outbreaks have primarily occurred in central to western Africa, sporadic outbreaks have been reported in other regions as a result of travel to endemic areas in Africa. In 2003, an outbreak was reported in Texas, United States, likely caused by the importation of an infected rodent [1,9].

Mpox usually develops after a latency period of 6-13 days (maximum 5-21 days) following viral exposure [10]. After the latent period, symptoms such as fever, headache, lymphadenopathy, and myalgia persist for approximately 1 to 5 days, followed by the development of a rash. The eruptions often appear on the face and extremities and gradually elevate, forming bullae, pustules, crusting, and eventually heal 2 to 4 weeks after onset. The fatality rate is reported to be 0%-11% and tends to be higher in children; however, no deaths have been reported in the developed nations [11,12].

Currently, tecovirimat is approved by the Food and Drug Administration for the treatment of mpox [13,14], but there is no approved medication for mpox in Japan. As a preprophylaxis measure, vaccination using the smallpox vaccine has been reported to be beneficial due to the significant overlap in immune response among viruses of the *Orthopoxvirus* genus (including smallpox virus, mpox, and vaccinia virus). Furthermore, based on the genomic analyses of mpox strains isolated in Europe, the mpox strains circulating since May 2022 more likely originate from the West African clade and exhibit a higher homology to smallpox virus strains [15]. An analysis of epidemiological data on mpox in the Republic of Zaire during the 5-year period from 1980 to 1984 suggests that smallpox vaccination prevents approximately 85% of mpox cases [16]. For postexposure prophylaxis, the Centers for Disease Control and Prevention recommends administration of the mpox vaccine for prophylaxis, ideally within 4 days of exposure, with vaccination within 14 days also being considered; however, the efficacy may be inferior after 4 days [17].

The smallpox vaccine has been classified into 3 generations by the WHO [18]. The first generation includes the Lister strain vaccine and Wyeth's Dryvax (NYCBH strain), which were manufactured and used during the smallpox eradication program by the WHO, contributing to smallpox eradication [19]. The second-generation smallpox vaccine uses the same smallpox vaccine strain as the first-generation vaccine and clonal viral variants purified from conventional vaccine strains, including ACAM2000 [20,21]. The third-generation smallpox vaccine is an attenuated vaccine strain specifically developed as a safer vaccine at the end of the eradication phase by cell culture and animal passage with LC16m8 strain vaccine and Bavarian Nordic's MVA-BN (JYNNEOS/Imvanex/IMVAMUNE) [22-24]. Among these, the third-generation LC16 is currently approved in Japan, although it is not widely distributed and cannot be used in general practice.

In this study, the close contacts of patients with mpox were vaccinated with the LC16, which is the approved smallpox vaccine in Japan, to determine the efficacy and safety of LC16 for postexposure prophylaxis against mpox.

## Methods

### Overview

This is a prospective, interventional, single-arm study aimed at evaluating the efficacy and safety of the LC16 smallpox vaccine for individuals who have had close contact with patients diagnosed with mpox.

**Figure 1:**
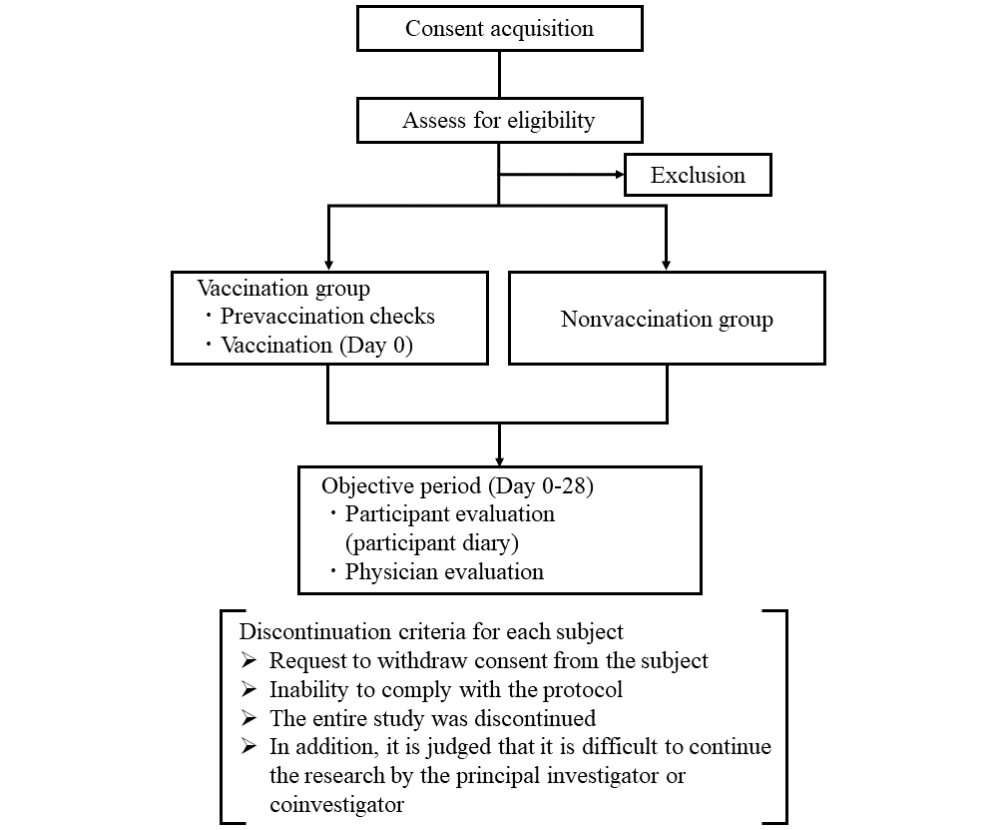
Overview of the study process.

The study was conducted according to the schedule described in Figure 1 and Table 1. Informed consent was obtained from the participants within 14 days after mpox exposure, which is defined as day 0. Only participants who provided consent to be vaccinated received the vaccine. Close contacts who did not wish to be vaccinated were included in the study for reference purposes and were observed for follow-up if they had consent to participate. Participants self-recorded their body temperature, presence of headache, rash, and lymphadenopathy daily for 21 days after vaccination (or vaccine refusal), as well as on day 28. On days 7, 14, 21, and 28 after vaccination (or vaccine refusal), the investigators or clinical research coordinator contacted the participants to inquire about any reported adverse events. The investigators also evaluated the onset of mpox 21 days after close contact (day 7 to 21). The definition of mpox onset is based on the notification of the Infectious Disease Law. In the cases of nonreserved visits or cancellations, the investigators confirmed the presence or absence of onset and any side effects. The primary end point includes the presence or absence of mpox 21 days after close contact. Secondary end points include postvaccination adverse reactions, the rate of severe disease, death, presence of symptoms (fever, headache, and lymphadenopathy), and complications (secondary skin infections, bronchopneumonia, sepsis, encephalitis, and keratitis).

The investigators decided whether to administer other live vaccines within 27 days. The investigators also explained to the participants that after inoculation with a vaccine, the participant may not be inoculated with other vaccines for a stipulated time period.

**Table 1:** Study time line.

Category		Time period (day)								
		–14 to –1	0	1 to 6	7 (allowable by ±2)	8 to 13	14 (allowable by ±2)	15 to 20	21 (allowable by ±2)	28 (allowable by ±2)
**Prevaccination** **checks**										
	Criteria	✓	—^a^	—	—	—	—	—	—	—
	Obtaining consent	✓	—	—	—	—	—	—	—	—
	Registration	✓	—	—	—	—	—	—	—	—
	Background	✓	—	—	—	—	—	—	—	—
	Concomitant medications	✓	—	—	—	—	—	—	—	—
Vaccination		—	✓^b^	—	—	—	—	—	—	—
Primary end point^c^		—	—	—	✓^d^	✓^d^	✓^d^	✓^d^	✓^d^	—
**Participant evaluation (participant diary)**										
	Body temperature	—	✓	✓	✓	✓	✓	✓	—	✓
	Headache	—	✓	✓	✓	✓	✓	✓	—	✓
	Rash	—	✓	✓	✓	✓	✓	✓	—	✓
	Lymphadenopathy	—	✓	—	—	—	—	—	—	—
**Physician evaluation**										
	Complication	—	—	—	✓	—	✓	—	✓	✓
	Any injection-site reaction (“take”)^e^	—	—	—	—	✓	✓	—	—	—

^a^Not applicable.

^b^Only participant to consent for vaccination.

^c^The primary end point is assessed through participant evaluation of symptoms (body temperature, headache, rash, and lymphadenopathy), as well as physician evaluation of complications and any local reactions at the injection site.

^d^Investigators evaluate the onset of mpox 21 days after close contact.

^e^Investigators evaluate any injection-site reaction (“take”) by examination or by reviewing a picture of the inoculation site.

### Study Setting

This study was conducted solely at the Center Hospital of the National Center for Global Health and Medicine (NCGM), Tokyo, Japan. NCGM is a 749-bed general hospital with close relationships to the Ministry of Health, Labour and Welfare (MHLW) and is a center for infectious disease treatment in Japan. In cases where a close contact is identified in an area far from Tokyo, a researcher from NCGM personally delivered the smallpox vaccine to the contact, obtained their consent for the study, and administered the vaccine if consent was granted. The MHLW has issued a notice on this policy and informed health centers throughout Japan. Close contacts cannot be vaccinated without participating in this study, indicating that although this is a single-center study, it encompasses the entire country of Japan.

### Eligibility Criteria

The eligibility criteria for this study are shown in Textbox 1.

The primary inclusion criteria are as follows: age of 1 year or older at the time of consent, participation within 14 days of close contact with an individual diagnosed with mpox, and no clear indication of mpox.

The main exclusion criteria are as follows: evident immunopathy, use of adrenocorticosteroids or immunosuppressants, history of anaphylactic shock caused by ingredients of the smallpox vaccine, and the presence of prevalent skin disease and potential disability by vaccination.

**Eligibility criteria.**

**Inclusion criteria**

- Provided written consent from the principal or proxy for participation
- Age of 1 year or older at the time of consent
- Participation within 14 days of close contact with an individual diagnosed with mpox. The definition of “close contact” refers to an individual who is determined to be a contact in an active epidemiological study conducted by the health center of the patient's address based on the criteria of an active epidemiological study issued by the Ministry of Health, Labour and Welfare
- No clear indication of mpox

**Exclusion criteria**

- Evident immunopathy
- Use of adrenocorticosteroids or immunosuppressants (such as cyclosporine, tacrolimus, and azathioprine)
- History of anaphylactic shock caused by ingredients of the smallpox vaccine
- Fever
- Presence of a serious acute disease
- Pregnancy
- Prevalent skin disease and potential disability by vaccination
- Not appropriate for vaccination
- Considered to be unfit to participate in this study by the principal investigators

### Informed Consent

Before obtaining informed consent, the investigators ensured that the participants were given sufficient time and opportunity to gather information about the study's details and make an informed decision regarding their participation. Participation in the study was voluntary, and participants could refuse to participate or withdraw from the trial at any time without penalty or loss of benefits to which the participant is otherwise entitled. The written informed consent was signed and dated by the participant and investigators. The explanatory and consent documents were approved by the authorizing clinical study examination committee and the MHLW.

### Interventions

The vaccine used in this study is a freeze-dried cell culture of smallpox vaccine LC16 (LC16m8 strain vaccine), manufactured by KM Biologics, Japan. It is a third-generation smallpox vaccine manufactured by cell culture of the attenuated virus strain [25]. The vaccine is dissolved in 0.5 mL of the attached solvent (20 vol% glycerylated water for injection) and inoculated into the skin with a bifurcated needle. The number of compressions at the site of vaccination is, as a guide, 5 for first-time inoculations and 10 for others.

### Criteria for the Discontinuation of Treatment or Intervention

The investigators review the continuation of the study when the following events occur: participant requests withdrawal of consent, inability to comply with the research protocol, discontinuation of the entire study, and decision of the investigator to conclude the study due to challenges in its continuation.

### Statistical Analysis

The study population (Full Analysis Set) excludes ineligible participants and those with missing data on efficacy end points among the enrolled participants. The study population for safety end point analysis is defined as the enrolled participants excluding nonvaccinated participants.

According to a systematic review on the epidemiology of mpox [26], 4 studies reported the incidence of mpox in nonvaccinated close contacts. Among individuals who were not vaccinated, 7.4% of close contacts developed mpox (92 cases out of 1238 close contacts). Therefore, for this study, we set the threshold for the incidence of mpox among nonvaccinated individuals at 7.4%. An older study also reported an incidence rate of 3 of 40 (7.5%) among family members who have been in close contact with the disease [27]. The incidence of mpox among vaccinated participants is calculated, and Bayes' theorem is used to determine the probability of the incidence being lower than 7.4% (posterior probability). Additionally, for the prior distribution of the vaccinated participants, we assume an uninformative distribution and determine that if the estimated posterior probability exceeds 90%, the vaccine has a certain effectiveness. In case no incidences of mpox occur, 33 cases within 4 days of close contact are required to obtain a 90% posterior probability.

Primary end point analysis will be performed among Full Analysis Set who had contact with an infected person within 4 days. The incidence rate and its 90% and 95% CI are calculated, as well as the posterior probability of the incidence rate less than 7.4%. For the secondary end points analysis, the severity rate, death rate, and their 95% CI for vaccinated cases are calculated. For the safety end points analysis, the number of adverse events and the incidence rate are calculated for each event using the safety population.

### Ethical Approval

This study was conducted in compliance with the ethical principles stipulated in the Declaration of Helsinki, the Clinical Research Act, relevant notices, and this research protocol. Ethical approval of the research protocol and the consent document have been obtained from the Certified Review Board of the National Center for Global Health and Medicine (NCGM-C-004504-02), and the study is registered in the Japan Registry of Clinical Trials (jRCTs031220137).

## Results

The first participant was enrolled on July 28, 2022. Recruitment and registration ended on December 16, 2022. Date fixation was completed in December 2022, and and the study was concluded in March 2023.

## Discussion

This is a study protocol to evaluate the efficacy and safety of the smallpox vaccine, LC16m8 strain vaccine, as a postexposure prophylaxis measure in individuals who have had close contact with patients with mpox.

Owing to changes in vaccine guidelines during the study, the number of compressions at the site of vaccination is, as a guide, considered as 15 [28]. The anticipated result of this study is to demonstrate the effectiveness of postexposure vaccination as a prophylaxis measure against mpox onset and severe disease. Specifically, the absence of symptoms (fever, headache, rash, and lymphadenopathy) listed in the diary recorded by the participant during the observation period after vaccination, and the presence of antibodies at the injection site (“take”) determined by the doctor's evaluation. These findings could provide new evidence for mpox treatment with postexposure vaccination recommended by the Centers for Disease Control and Prevention.

There are some limitations to this study. First, this study had to be conducted in a short period of time owing to the rapid spread of the epidemic, and we could not afford to design a multicenter study. However, because the researchers visited participants residing in distant areas, the study can be considered to cover the whole of Japan, thus ensuring its validity. In addition, this study is open-labeled as there was insufficient time to establish a blinding system and prepare a placebo. While it is impossible to completely eliminate bias in the evaluations conducted by investigators and participants, there are objective criteria for the diagnosis of mpox; therefore, bias in the primary end point can be eliminated. Additionally, although 2 groups (vaccinated and nonvaccinated) have been formed, they were not randomized, rather participants decided whether to be vaccinated or not based on their personal preferences. Comparison between the 2 groups is challenging since this is a nonrandomized study. However, the efficacy is evaluated based on the reported incidence of mpox.

In conclusion, this is an ongoing, open-label trial, and observing the incidence of mpox in the vaccine group will provide the most definitive efficacy comparison data and other important clinical outcome data to date. If the incidence of mpox is below 7.4% in the vaccination group and the hypothesis that the LC16m8 strain vaccine is safe is valid, the LC16m8 strain vaccine would be an important treatment option for individuals in close contact with patients diagnosed with mpox. This outcome could lead to the swift integration of the LC16m8 strain vaccine into clinical practice.

## Abbreviations

MHLW: Ministry of Health, Labour and Welfare
NCGM: Center Hospital of the National Center for Global Health and Medicine
WHO: World Health Organization

## Data Availability

There are no plans to share data. As the possibility of secondary data use is explained in the consent document, secondary use of data is possible as long as a new research protocol is prepared, reviewed, and approved by the appropriate review committee.

## References

[1] Parker S, Buller RM (2013). A review of experimental and natural infections of animals with monkeypox virus between 1958 and 2012. Future Virol.

[2] (2022). WHO recommends new name for monkeypox disease. World Health Organization.

[3] Petersen E, Kantele A, Koopmans M, Asogun D, Yinka-Ogunleye A, Ihekweazu C, Zumla A (2019). Human monkeypox: epidemiologic and clinical characteristics, diagnosis, and prevention. Infect Dis Clin North Am.

[4] Patel A, Bilinska J, Tam JCH, Da Silva Fontoura D, Mason CY, Daunt A, Snell LB, Murphy J, Potter J, Tuudah C, Sundramoorthi R, Abeywickrema M, Pley C, Naidu V, Nebbia G, Aarons E, Botgros A, Douthwaite ST, van Nispen Tot Pannerden C, Winslow H, Brown A, Chilton D, Nori A (2022). Clinical features and novel presentations of human monkeypox in a central London centre during the 2022 outbreak: descriptive case series. BMJ.

[5] Lourie B, Bingham PG, Evans HH, Foster SO, Nakano JH, Herrmann KL (1972). Human infection with monkeypox virus: laboratory investigation of six cases in West Africa. Bull World Health Organ.

[6] Ladnyj ID, Ziegler P, Kima E (1972). A human infection caused by monkeypox virus in Basankusu territory, democratic Republic of the Congo. Bull World Health Organ.

[7] Hutin YJ, Williams RJ, Malfait P, Pebody R, Loparev VN, Ropp SL, Rodriguez M, Knight JC, Tshioko FK, Khan AS, Szczeniowski MV, Esposito JJ (2001). Outbreak of human monkeypox, democratic Republic of Congo, 1996 to 1997. Emerg Infect Dis.

[8] Grant R, Nguyen LBL, Breban R (2020). Modelling human-to-human transmission of monkeypox. Bull World Health Organ.

[9] Kumar N, Acharya A, Gendelman HE, Byrareddy SN (2022). The 2022 outbreak and the pathobiology of the monkeypox virus. J Autoimmun.

[10] (2022). Mpox (monkeypox). World Health Organization.

[11] Sklenovská N, Van Ranst M (2018). Emergence of monkeypox as the most important orthopoxvirus infection in humans. Front Public Health.

[12] Jezek Z, Khodakevich LN, Wickett JF (1987). Smallpox and its post-eradication surveillance. Bull World Health Organ.

[13] Sherwat A, Brooks JT, Birnkrant D, Kim P (2022). Tecovirimat and the treatment of monkeypox—past, present, and future considerations. N Engl J Med.

[14] Matias WR, Koshy JM, Nagami EH, Kovac V, Moeng LR, Shenoy ES, Hooper DC, Madoff LC, Barshak MB, Johnson JA, Rowley CF, Julg B, Hohmann EL, Lazarus JE (2022). Tecovirimat for the treatment of human monkeypox: an initial series from Massachusetts, United States. Open Forum Infect Dis.

[15] Giorgi FM, Pozzobon D, Meglio AD, Mercatelli D (2022). Genomic analysis of the recent monkeypox outbreak. bioRxiv.

[16] Fine PE, Jezek Z, Grab B, Dixon H (1988). The transmission potential of monkeypox virus in human populations. Int J Epidemiol.

[17] (2022). Vaccination. CDC.

[18] (2022). Vaccines and immunization for monkeypox: interim guidance, 16 November 2022. World Health Organization.

[19] Jezek Z, Marennikova SS, Mutumbo M, Nakano JH, Paluku KM, Szczeniowski M (1986). Human monkeypox: a study of 2,510 contacts of 214 patients. J Infect Dis.

[20] Weltzin R, Liu J, Pugachev KV, Myers GA, Coughlin B, Blum PS, Nichols R, Johnson C, Cruz J, Kennedy JS, Ennis FA, Monath TP (2003). Clonal vaccinia virus grown in cell culture as a new smallpox vaccine. Nat Med.

[21] Nalca A, Zumbrun EE (2010). ACAM2000: the new smallpox vaccine for United States Strategic National Stockpile. Drug Des Devel Ther.

[22] (2018). Identifying and responding to serious adverse events following immunization, following use of smallpox vaccine during a public health emergency: a guidance document for smallpox vaccine safety surveillance. World Health Organization.

[23] Greenberg RN, Overton ET, Haas DW, Frank I, Goldman M, von Krempelhuber A, Virgin G, Bädeker N, Vollmar J, Chaplin P (2013). Safety, immunogenicity, and surrogate markers of clinical efficacy for modified vaccinia Ankara as a smallpox vaccine in HIV-infected subjects. J Infect Dis.

[24] Volkmann A, Williamson AL, Weidenthaler H, Meyer TPH, Robertson JS, Excler JL, Condit RC, Evans E, Smith ER, Kim D, Chen RT, Brighton Collaboration Viral Vector Vaccines Safety Working Group V3SWG (2021). The brighton collaboration standardized template for collection of key information for risk/benefit assessment of a Modified Vaccinia Ankara (MVA) vaccine platform. Vaccine.

[25] Kenner J, Cameron F, Empig C, Jobes DV, Gurwith M (2006). LC16m8: an attenuated smallpox vaccine. Vaccine.

[26] Beer EM, Rao VB (2019). A systematic review of the epidemiology of human monkeypox outbreaks and implications for outbreak strategy. PLoS Negl Trop Dis.

[27] Breman JG, Steniowski MV, Zanotto E, Gromyko AI, Arita I, Kalisa-Ruti (1980). Human monkeypox, 1970-79. Bull World Health Organ.

[28] (2022). Package insert of the LC16m8 vaccine.

